# Virilization at puberty in adolescent girls may reveal a 46,XY disorder of sexual development

**DOI:** 10.1530/EC-23-0267

**Published:** 2023-11-08

**Authors:** A Bergougnoux, L Gaspari, M Soleirol, N Servant, S Soskin, S Rossignol, K Wagner-Mahler, J Bertherat, C Sultan, N Kalfa, F Paris

**Affiliations:** 1 Service de Génétique Moléculaire et de Cytogénétique, Centre Hospitalier Universitaire de Montpellier, Université de Montpellier, Montpellier, France; 2 Département d'Endocrinologie et de Gynécologie Pédiatrique, Hôpital Arnaud de Villeneuve, Université de Montpellier, Montpellier, France; 3 INSERM Unité 1203 (DEFE), Université de Montpellier, Montpellier, France; 4 Département de Pediatrie, CHU Nîmes, France, Université de Montpellier Faculté de Médecine Montpellier-Nîmes, Montpellier, France; 5 Département de Pédiatrie, Centre Hospitalier Universitaire Hautepierre de Strasbourg, Strasbourg, France; 6 Département de Pédiatrie, CHU Nice, Hôpitaux Pédiatriques de Nice CHU-Lenval, Nice, France; 7 Department of Endocrinology, French Reference Center for Rare Adrenal Disorders, Hôpital Cochin, Université Paris Cité, Institut Cochin, Assitance Publique-Hôpitaux de Paris, Paris, France; 8 Department of Pediatric Urological Surgery, French Reference Center for abnormalities of Genital Development (DevGen), CHU Lapeyronie, Montpellier University, Montpellier, France

**Keywords:** 46,XY DSD, pubertal virilization, 46,XY sex reversal

## Abstract

Although hyperandrogenism is a frequent cause of consultation in adolescent girls, more severe forms with virilization must lead to suspicion of an adrenal or ovarian tumor. However, they may also reveal a 46,XY disorder of sexual development (DSD). Here, we describe four adolescent girls referred for pubertal virilization and in whom we diagnosed a 46,XY DSD. We performed gene mutation screening by Sanger sequencing (all patients) and by next-generation sequencing (NGS) in patient #4. We identified new heterozygous* NR5A1* gene variants in patients #1 and #2 and a homozygous *SRD5A2* gene deletion in patient #3. Patient #4 received a diagnosis of complete androgen insensitivity in childhood; however, due the unusual pubertal virilization, we completed the gene analysis by NGS that revealed two heterozygous *HSD17B3* variants. This work underlines the importance of considering the hypothesis of 46,XY DSD in adolescent girls with unexplained virilization at puberty.

## Introduction

Mild/moderate hyperandrogenism is frequent in adolescent girls (moderate acne) and is often associated with irregular menstrual cycles. It is most often transient and related to the hypothalamic–pituitary–ovary axis immaturity during this period and will decrease/disappear in the first years following menarche (1). In some girls, clinical signs of hyperandrogenism may persist after 2 years post menarche and worsen (hirsutism) (2, 3). These persistent and severe forms of hyperandrogenism warrant investigations to rule out an adrenal or ovarian disorder, particularly polycystic ovary syndrome and nonclassical congenital adrenal hyperplasia (2, 3). In few patients, the clinical expression of hyperandrogenism is more severe with rapidly worsening hirsutism and other signs of virilization, such as clitoromegaly, voice deepening and muscle mass increase. This must suggest an adrenal or ovarian tumor (2, 3). In all patients, the workup should include at least the quantification of testosterone and gonadotropins in plasma. Moreover, quantification of plasma 17-hydroxyprogesterone, and possibly of other androgens (e.g. delta-4 androstenedione (A4) and dehydroepiandrosterone sulfate (DHEA-S)), and abdominopelvic ultrasonography are useful to identify the hyperandrogenism cause. Independently of the presence or not of Müllerian structures, karyotyping must also be performed to exclude a 46,XY disorder of sex development (DSD) resulting in sex reversal. Indeed, in the absence of any particular warning sign (e.g. inguinal gonads or neonatal clitoral enlargement), 46,XY individuals with severe undervirilization (i.e. with normal female external genitalia) are unsurprisingly identified and reared as girls from birth. In these patients, 46,XY DSD will only be diagnosed at puberty because of amenorrhea and/or virilization. Among the various causes of 46,XY DSD, 5α-reductase type 2 (encoded by *SRD5A2*) (4, 5, 6, 7, 8) or 17β-hydroxysteroid dehydrogenase type III (encoded by *HSD17B3*) deficiency (9, 10, 11) and alteration of steroidogenic factor 1 (SF-1; encoded by *NR5A1*) function (12, 13, 14, 15, 16, 17, 18, 19, 20) have been associated with virilization at puberty. Through the description of four cases, we wanted to demonstrate that virilization in adolescent girls (i) may reveal a 46,XY DSD and (ii) requires a precise genetic evaluation.

## Patients and methods

### Patients

The expertise of the DevGen (genital development) team of Montpellier University Hospital, France, includes the medical (pediatric/adult endocrinology services) and surgical management of patients with DSD and also the molecular study of these disorders. In the past 20 years, we analyzed more than 1500 DNA samples from patients with 46,XY DSD followed by our service and by other French teams. In addition, we followed more than 900 patients with complex 46,XY DSD in our clinical practice in the past 20 years.

The present study focused on the clinical, biochemical, and molecular characterization of four patients with pubertal virilization and 46,XY DSD diagnosed at our center between 2002 and 2022. The only other case of authentic pubertal virilization in an adolescent girl, referred to our pediatric endocrinology department during this period, was an adolescent 46,XX girl with adrenocortical cancer. The study was approved by Montpellier University Hospital Ethics Committee. Written informed consent was obtained from all four patients.

Pubertal development was assessed using the pubic hair (P) and breast (B) development stages 1–5 of the Tanner scale (21). Hirsutism was quantified using the modified Ferriman–Gallwey (FG) scoring system that includes nine androgen-sensitive body areas (22). All hormone reference values are reported in Table 1 (23, 24).

**Table 1:** Reference values of the indicated hormones in females and males at Tanner 4–5 (23, 24), reference values from our laboratory for AMH and inhibin B.

	Women	Men
Testosterone (nmol/L)	0.5–2.0	7.6–27.0
DHT (nmol/L)	0.1–0.7	0.2–3.2
∆4 (nmol/L)	1.7–10.0	1.5–6.4
DHEA-S (µmol/L)	1.3–11.1	2.8–15.3
FSH (IU/L)	3.5–12.5	1.5–12.4
LH (IU/L)	2.4–12.6	1.7–8.6
AMH (ng/mL)	0.3–6.3	1.3–46.5
Inhibin B (pg/mL)	10–150	60–300

∆4, delta-4 androstenedione; AMH, anti-Müllerian hormone; DHEA-S, dehydroepiandrosterone sulfate; DHT, dihydrotestosterone; FSH, follicle-stimulating hormone; LH, luteinizing hormone.

Patient #1 was a 17-year-old girl referred to the pediatric endocrinology service because of primary amenorrhea. At the first consultation, the pubertal status was B1P5 (Tanner scale) and a clear clinical hyperandrogenism was noted: hirsutism (FG score = 18), acne, and clitoromegaly. The plasma concentrations of testerosterone, follicle-stimulating hormone (FSH), and luteinizing hormone (LH) were 10.4 nmol/L, 58.3 IU/l, and 16.4 IU/L, respectively. The karyotype was 46,XY. MRI showed the presence of a Müllerian duct remnant of 27 mm × 15 mm not visible on ultrasonography. Surgery was performed to remove the gonads due to testicular dysgenesis highlighted by the hormonal workup. The anatomopathological study confirmed partial testicular dysgenesis and showed also the presence of a right gonadal seminoma. The clinical and biological hyperandrogenism regressed after gonadectomy.

Patient #2 was referred to the pediatric endocrinology service at the age of 14 years due to clinical hyperandrogenism. At clinical examination, height was 176 cm, weight was 56 kg, without breast development, the pubertal status was B1P5 (Tanner scale), but with important hirsutism (FG score = 20). The gynecological examination found clitoris enlargement (60 mm). The plasma concentrations of testerosterone, FSH, and LH were 21.9 nmol/L, 27.3 IU/L, and 9.2 IU/L, respectively. Anti-Müllerian hormone (AMH) and inhibin B concentrations in blood were 0.49 ng/mL and 5 pg/mL, respectively. The karyotype was 46,XY. MRI showed the presence of a prepubertal uterus (33 mm in height) and two pelvic gonads.

Patient #3 reported virilization at the age of 12 years with hirsutism (FG score = 15), gradual voice deepening, and development of the genital tubercule that bothered the patient who started gender reassignment at the age of 17 years. This patient of Iraqi origin came to France at the age of 18 years and was referred to the endocrine department. The clinical examination showed absence of breast development, the pubertal status was B1P5 (Tanner scale), hirsutism (FG score = 20), clitoromegaly (35 mm), and a palpable left inguinal gonad (the right gonad might have been removed during childhood following a trauma, without any tissue or genetic analysis). The plasma concentrations of testerosterone, dihydrotestosterone (DHT), A4, and DHEA-S were 19.8 nmol/L, 0.3 nmol/L, 7.9 nmol/L, and 9.4 μmol/L, respectively. They were associated with elevated FSH and LH concentrations: 33.1 IU/L and 10.3 IU/L. AMH and inhibin B concentrations were 4.5 ng/mL and 16 pg/mL, respectively. The karyotype was 46,XY. The parents were first cousins. Ultrasonography and pelvic MRI did not find any Müllerian duct remnant.

Patient #4 was followed by the pediatric endocrinology service unit of the DevGen team of Montpellier for complete androgen insensitivity syndrome (CAIS) diagnosed at the age of 10 years in the context of bilateral inguinal hernia associated with palpable inguinal gonads. At that time, the pubertal status was B1P1 (Tanner scale). Ultrasonography confirmed the presence of inguinal gonads but did not find the uterus. The karyotype was 46,XY. The plasma concentrations of testerosterone, FSH, LH, AMH, and inhibin B were <0.87 nmol/L, 1.7 IU/L, 1.3 IU/L, 516 ng/mL, and 277 pg/mL, respectively. Sanger sequencing of the androgen receptor (*AR*) gene identified the c.2395C>G, p.(Gln799Glu) variant that was previously reported in patients with partial and mild androgen insensitivity (McGill University database). The mother was a heterozygous carrier of the same mutation. At the age of 14 years, the patient complained of hirsutism and acne, as well as of feeling lumps in the inguinal area. At clinical examination, the height was 169 cm, weight 49.5 kg, and pubertal status B3P5 (Tanner scale). Hirsutism was marked on the face and inner thighs (FG score = 15), associated with acne on the face and back, and deepened quality of voice. Gynecological examination found clitoromegaly (50 mm). The plasma concentrations of testerosterone, FSH, LH, A4, AMH, and inhibin B were 20.8 nmol/L, 7.8 IU/L, 35.2 IU/L, 40.2 nmol/L, 78.55 ng/mL, and 379.9 pg/mL, respectively.

The patients’ phenotypes are summarized in Table 2.

**Table 2:** Patients’ phenotypes.

Patient	Age at referral/virilization (years)	Phenotype at puberty	Müllerian structures on US/MRI	Testosterone level (nmol/L)	Gonadotropins (IU/L)	AMH (ng/ mL)	Inhibin B (pg/mL)	Other hormones, if available	Comments	Pathology analysis in case of gonadectomy
1	17	B1P5, primary amenorrhea, hirsutism (FG 18), acne, clitoromegaly	Yes	10.4	LH: 16.4, FSH: 58.3	ND	ND			Partial testicular dysgenesis, Right gonadal seminoma
2	14	B1P5, hirsutism (FG 20), clitoris enlargement (60 mm)	Yes	21.9	LH 9.2, FSH: 27.3	0.49	5			
3	18	B1P5, hirsutism (FG 20), gradual onset of voice deepening, clitoromegaly (35 mm), palpable left inguinal gonad	No	19.8	LH: 10.3, FSH: 33.1	4.5	16	DHT: 0.3 nM, ∆4: 7.9 nM, DHEA-S: 9.4 µM		
4	14	B3P5, hirsutism (FG 15), acne face/back, raucous voice, clitoromegaly (50 mm)	No	20.8	LH: 35.2, FSH: 7.8	78.5	379.9	∆4: 40.2 nM	At the age of 10 years, the discovery of a bilateral inguinal hernia with palpable inguinal gonads led to the diagnosis of complete androgen insensitivity syndrome (CAIS). The *AR* gene mutation was transmitted by the mother	

## Methods

Hormone concentrations were measured by independent laboratories.

Genomic DNA was extracted from peripheral blood leucocytes using the QIAamp DNA blood mini-kit (Qiagen) following the manufacturer’s instructions. As previously reported, exons 1–5 of the *SRD5A2* gene (25) and exons 2–7 of the *NR5A1* gene (26) were amplified by PCR, and direct sequencing was performed with the BigDye terminator version 1.1 kit (Applied Biosystems) and an ABI Prism 3130 genetic analyzer (Applied Biosystems).

For patient #4, the unusual observation of pubertal virilization in the context of CAIS led to complete the genetic analysis by next-generation sequencing (NGS) of genes implicated in DSD. Mutations in these genes have been associated with sexual determination/differentiation alterations, hypogonadotropic hypogonadism, and steroidogenesis alteration. The implication of these genes has been validated in human diseases or is suspected because of the results of knockout and/or knockdown experiments showing repercussions on sex determination or differentiation in animal models. Massive Parallel Sequencing libraries were prepared following the manufacturer’s instructions. Briefly, DNA libraries were prepared using SeqCap EZ probes, the KAPA HyperPrep and HyperCap Target Enrichment Kits (Roche Diagnostics®). Libraries were sequenced in independent runs using a MiniSeq High Output Reagent Cartridge and Flowcell on a MiniSeq (Illumina, San Diego, CA, USA). Samples reached high quality control levels. Target coverage at 50× was 99.0%. Reads were aligned using Local Run Manager (Illumina) or the MobiDL workflow developed by the MoBiDiC team (https://github.com/mobidic/MobiDL). This workflow is dedicated to NGS data obtained using SeqCap libraries and focuses on gene panels/exomes. It uses the Genome Analysis ToolKit (GATK) 4 HaplotypeCaller and Google DeepVariant for variant calling. Copy number variants were detected using the MobiCNV tool (https://github.com/mobidic/MobiCNV). The Captain Achab workflow and the MoBiDiC prioritizing algorithm (27) were used for single-nucleotide variant filtering and prioritization. The pathogenicity of rare variants was predicted by the MoBidetails Online DNA Variant Interpretation tool that exploits multiple sources (https://mobidetails.iurc.montp.inserm.fr/MD/). The suspected disease-causing *HSD17B3* variants were confirmed by Sanger sequencing with the following primers: exon 3 forward 5′-TAACACAAGCCCTCCCTGTC-3′; reverse 5′-GAGCAGGCTTGGTTGGAG-3′; exon 4 forward 5′-GGGCATTTGGATCCCTG-3′; reverse 5′-CATCAGTGTCAGGTTATTTCACTG-3′.

## Results

Sequencing allowed identification of two new heterozygous *NR5A1* gene variants, c.1069C>T, p.(Gln357Ter) in patient #1 and c.38G>C, p.(Cys13Ser) in patient #2; a new homozygous deletion of one base pair in *SRD5A2*, c.453del, p.(Leu152TyrfsTer8) in patient #3; and two compound heterozygous variants of the *HSD17B3* gene, c.277+4A>T, p.(?) (inherited from the father) and c.278-1G>C, p.(?) (inherited from the mother) in patient #4. Both variants are recurrent variants in patients with 17β-hydroxysteroid dehydrogenase type III deficiency (28). They were confirmed (c.277+4A>T) or predicted (c.278-1G>C) to disrupt canonical splicing sites, leading to aberrant splicing and to the occurrence of premature stop codons inside exon 4.

All variants detected in the four patients and their interpretation according to the American College of Medical Genetics and Genomics (ACMG) guidelines and standards are reported in Table 3.

**Table 3:** Gene variants.

Patient	Gene RefSeq transcript	Variant c	Variant p	Frequency gnomAD V3.1.2	ClinVar	Status	ACMG classification (46)	Predicted effect/functional studies
1	*NR5A1* NM_004959.5	c.1069C>T	p.(Gln357Ter)	No match	No match	Heterozygous	PVS1 PM2 → Likely pathogenic	Premature stop codon
2	*NR5A1* NM_004959.5	c.38G>C	p.(Cys13Ser)	No match	No match	Heterozygous	PS2 PM1 PM2 PP3 → Likely pathogenic	Missense affecting Zinc Finger domain 1
3	*SRD5A2* NM_000348.3	c.453del	p.(Leu152TyrfsTer8)	No match	No match	Homozygous	PVS1 PM2 PM3 → Pathogenic	Reading frame shift with premature stop in exon 3
4	*HSD17B3* NM_000197.2	c.277+4A>T	p.(?)	0.00035	Pathogenic	Heterozygous (inherited from the father)	PVS1 PM2 PM3 → Pathogenic	Skipping of exon 3, frameshift with premature stop codon in exon 4 (23)
4	*HSD17B3* NM_000197.2	c.278-1G>C	p.(?)	0.00003	Pathogenic	Heterozygous (inherited from the mother)	PVS1 PM2 PM3 → Pathogenic	Acceptor native site loss, predicted use of cryptic acceptor site in exon 3, frameshift with premature stop codon in exon 3
4	*AR* NM_000044.6	c.2395C>G	p.(Gln799Glu)	0.0013	Conflicting interpretation of pathogenicity	Hemizygous	PM1 PP3 PP5 BS1 BP5 → Probably benign	Missense, effect on the transactivation capacity of androgen receptor (32)

## Discussion

Here, we described four cases of adolescent girls with virilization in whom the clinical, laboratory, and sequencing analyses led to the diagnosis of 46,XY DSD caused by *NR5A1* (patients #1 and #2), *SRD5A2* (patient #3), and *HSD17B3* variants (patient #4; initial diagnosis of CAIS).

*NR5A1* alterations are associated with a large spectrum of clinical phenotypes, from isolated infertility in male patients to 46,XY sex reversal, without adrenal failure in most patients (29, 30). In the literature, there are only nine reports on pubertal virilization revealing 46,XY DSD in patients who were identified as females at birth (12, 13, 14, 15, 16, 17, 18, 19, 20). We and others previously reported that pubertal virilization (19) and also isolated primary amenorrhea (31, 32) should orient towards a diagnosis of 46,XY sex reversal related to *NR5A1* alterations (MIM #612965).

Different hypotheses have been proposed to explain the paradox of androgen production failure during the fetal male sexual differentiation period and recovery of testicular androgen production at puberty. Specifically, fetal Leydig cell (FLC) steroidogenesis could be more SF-1-dependent than adult Leydig cell (ALC) steroidogenesis (33). It is currently acknowledged that FLC and ALC are different cell populations (33, 34). In 2015, Karpova *et al.* demonstrated in male mice, that SF-1 is needed for the proper development of FLC and ALC, but with distinct functions: cell differentiation in FLC and progenitor cell formation and/or survival in ALC (35). However, Shima *et al.* reported that most ALC derive from dedifferentiated FLC and that *NR5A1* is essential for the initial FLC differentiation and also for ALC redifferentiation at puberty (36). Besides SF-1 essential role in FLC and ALC, ALC development involves many other transcription factors, growth factors, luteinizing hormone and androgens (37). Therefore, it could be hypothesized that some of these factors may partly compensate for the SF-1 defect, particularly at puberty, when luteinizing hormone signaling is critical for ALC differentiation and precursor proliferation. Moreover, in the adult testis, liver receptor homolog-1 (LRH-1), also known as nuclear receptor subfamily 5, group A, member 2 (NR5A2; a totipotency pioneer factor), could replace SF-1 to regulate steroidogenesis. Indeed, it is abundantly expressed in ALC, but not in FLC (38). In addition, FLC cannot convert A4 to testosterone. This is done by Sertoli cells, the only cells that express 17β-hydroxysteroid dehydrogenase type III in fetal testes. Therefore, impaired Sertoli cell function in patients with pathogenic *NR5A1* variants may be implicated in the more severe testosterone defect observed in fetal life compared with adult life (33). Indeed, the increase of testosterone production at puberty is preserved because of the shift in 17β-hydroxysteroid dehydrogenase type 3 production from fetal Sertoli cells to ALC (33).

Patient #3 had a 5α-reductase type 2 deficiency (MIM #264600). Our group and others have described the large clinical phenotype variability of patients with 5α-reductase enzyme deficiency (4, 5, 39). Nevertheless, we clearly demonstrated that severe undervirilization is the most frequent clinical sign, as indicated by the finding that >72% of patients are identified as females at birth (5). We (4, 5, 6) and others (7) showed that patients with 46,XY DSD and sex reversal present almost systematically some degree of pubertal virilization, associated with disease-causing compound heterozygous or homozygous *SRD5A2* variants. In these patients, virilization is explained by the rise in serum testosterone at puberty, which may be metabolized to dihydrotestosterone by the type 1 isoenzyme that is expressed in skin from puberty. Our previous study found that 9.1% of patients from our cohort (total *n* = 55) requested female-to-male reassignment (5), as done by patient #3. On the basis of the better knowledge on the consequences of 5α-reductase type 2 deficiency in 46,XY patients and the increased success rates of assisted reproduction treatments in individuals with less severe undervirilization, currently, we favor male sex rearing from birth in this condition (7).

Patient #4 presented 17β-hydroxysteroid dehydrogenase type 3 deficiency (MIM #264300), a well-known cause of 46,XY DSD with sex reversal and virilization at puberty (11). At puberty, the isoenzyme 17β-hydroxysteroid dehydrogenase type 5 can convert ∆4 to testosterone (10). However, our initial diagnosis (at the age of 10 years) was CAIS related to the presence of the *AR* variant c.2395C>G, p.(Gln799Glu), inherited from the mother. This variant is located in the ligand binding domain of AR, and has a frequency of 1.3 × 10^−3^ in the general population (gnomAD V3). It has been detected in patients with less severe phenotype, ranging from mild androgen insensitivity syndrome with infertility (40, 41) to partial androgen insensitivity syndrome (42, 43). To our knowledge, only one study reported the association in the same patient with CAIS of this variant and another *AR* gene variant (44). *In vitro* studies reported impaired AR transactivation without binding (40, 41, 42) or *N*- and *C*-terminal domain interaction alteration (45). This variant remains of uncertain significance according to the ACMG classification (PM1, PP3, PP5, BS1). However, in our patient, it might have contributed to the fetal undervirilization and less severe virilization at puberty. Moreover, this patient demonstrates that any clinical sign or disease progression discordant with a previous molecular diagnosis should lead to the reevaluation of the etiological diagnosis and to more in-depth genetic analyses.

In conclusion, this work demonstrates that it is important to consider the hypothesis of a 46,XY DSD in adolescent girls with unexplained pubertal virilization. This clinical presentation warrant prompt investigations to exclude the presence of an adrenal or ovarian tumor. Karyotyping should be performed, independently of the presence/absence of Müllerian structures. Genetic analyses, including most genes previously described in 46,XY DSD, are required to establish the precise molecular diagnosis, to optimize patient management, and to propose genetic counseling.

## Declaration of interest

The authors declare that there is not conflict of interest that could be perceived as prejudicing the impartiality of the study reported.

## Funding

This work has not received any funding.
